# Hospital anxiety and depression scale cutoff scores for cancer patients in acute care

**DOI:** 10.1038/sj.bjc.6604952

**Published:** 2009-02-24

**Authors:** S Singer, S Kuhnt, H Götze, J Hauss, A Hinz, A Liebmann, O Krauß, A Lehmann, R Schwarz

**Affiliations:** 1 Department of Epidemiology and Population Health, London School of Hygiene and Tropical Medicine, University of London, London, UK; 2 Department of Social Medicine, University of Leipzig, Leipzig, Germany; 3 Department of Surgery, University of Leipzig, Leipzig, Germany; 4 Department of Medical Psychology, University of Leipzig, Leipzig, Germany; 5 Department of Radiology, University of Leipzig, Leipzig, Germany; 6 Clinic for Psychiatry, Psychosomatics and Psychotherapy, Parkkrankenhaus Leipzig-Südost, Leipzig, Germany

**Keywords:** diagnosis, in-patients, mental health, neoplasms, sensitivity and specificity

## Abstract

The aim of this study was to determine optimal cutoff scores for the Hospital Anxiety and Depression Scale (HADS) when used in evaluating cancer patients in acute care. A total of 689 cancer patients were assessed during their first days of in-patient treatment, using the structured clinical interview for DSM and the HADS. Statistical analysis was performed using ROC curves. A total of 222 patients (32%) had a mental disorder. The area under the curve was the best in the total scale of the HADS, namely 0.73. With a score of ⩾13, it is possible to detect 76% of the cases with a specificity of .60, whereas 95% of the cases can be detected with a score of ⩾6 (specificity 0.21). With scores of ⩾16 and ⩾22, recommended by the test authors for primary care, only 59 and 30% of the comorbid cancer patients are indicated. Lower HADS cutoff scores when preferable when evaluating cancer patients than are recommended for use in primary care. When using HADS in clinical practice and epidemiological studies, it is important to decide whether, for the task at hand, high detection rates of affected patients or low misclassification rates are more important.

As cancer is a potentially life-threatening disease, usually involving relatively aggressive and intrusive treatment, it presents a challenge for the patients' psychological adjustment and ability to cope. Comprehensive care, therefore, includes not only medical procedures but also mental health care. As a prerequisite, it is important to identify patients who are mentally affected and need further support and/or treatment.

On the one hand, it is known that a third of all cancer patients receiving oncological treatment, and half of the patients who have advanced cancer, suffer from mental distress, to an extent that they can be diagnosed with a psychiatric disorder (Derogatis *et al*, 1983; Härter *et al*, 2000; Atesci *et al*, 2004; Miovic and Block, 2007; Singer *et al*, 2007; Walker *et al*, 2007). On the other hand, physicians and nurses often fail to identify distressed patients (Newell *et al*, 1998; Fallowfield *et al*, 2001; Söllner *et al*, 2001; Keller *et al*, 2004), resulting in under-treatment in 40–90% of the cases (Flatten *et al*, 2003; Singer *et al*, 2005). It is, nevertheless, possible to improve the detection rates and treatment with intensive training (Passik *et al*, 2002; Adkins *et al*, 2005). Feedback of patients' self-reported psychosocial well-being to the oncologist alone did not, however, reduce the levels of anxiety and depression in a randomised controlled trial (Boyes *et al*, 2006).

Therefore, questionnaires screening for psychological comorbidity and cooperation with mental health care professionals are the most usual way to improve detection rates and patient care. Several indices have been developed and validated so far, and the debate regarding the question of which tool is the most suitable for the purpose is ongoing (Kirsh *et al*, 2004; Herschbach, 2006).

In oncological contexts, the HADS is one of the most commonly used questionnaires for identifying distress (Zigmond and Snaith, 1983). This is a 14-item questionnaire with cutoff points indicating ‘cases’ helping oncologists to decide whether or not a mental health care professional should be integrated in the treatment of a patient – an advantage in day-to-day care.

Each item of the HADS has a Likert response scale. Scores are constructed by summation, whereby increasing scores indicate increasing burden. There are two subscales: depression (HADS-D) and anxiety (HADS-A).

The optimal cutoff point is said to be ⩾8 for the identification of suspicious cases and ⩾11 for safe cases on both subscales (Zigmond and Snaith, 1983; Bjelland *et al*, 2002), with a sensitivity and specificity of 0.80 on an average (Bjelland *et al*, 2002). These scores, however, were conceived for evaluating primary care patients. It has been shown (Morse *et al*, 2005) that these thresholds might be too high for cancer patients, resulting in underrecognition of ‘cases’. Therefore, lower thresholds may be required without necessarily compromising specificity. In addition, it would be easier to handle one score instead of two in clinical routine; therefore, we wanted to test the performance of the HADS total scale with regard to specificity and sensitivity.

However, what is an optimal cutoff point? Some authors prefer a score that (a) gives the best proportion of sensitivity and specificity, indicating the point at which the percentage of wrongly classified patients is reduced to a minimum (‘balanced score’). However if, as should be the case in day-to-day care, it is deemed less desirable for a patient with problems to go undiagnosed and in continued need of psychosocial help than it is for a psycho-oncologist to be called ‘unnecessarily’ to talk with a mentally healthy patient, we need another score termed the (b) ‘clinical score’, defined as being a point at which at least 95% of cases are identified. For research purposes, it can also be interesting to define another score called the (c) ‘specific score’, whereby specificity is 0.95, indicating that 95% of the identified cases are true cases. With this study, we tried to find these three cutoff scores for a large number of cancer in-patients and compare them to the scores suggested by the authors of the HADS.

## Study design and procedure

We conducted a multicentre cross-sectional study with consecutive patient accrual. Study clinics were clinics of the University of Leipzig (Department of Surgery II, Department of Obstetrics and Gynaecology, Department of Urology and Department of Radiotherapy) and Leipzig's St Georg Hospital's Department of Obstetrics and Gynaecology.

Inclusion criteria were cancer diagnosis, age 18 or above, ability to speak and understand German, ability to complete a questionnaire and informed consent.

All in-patients diagnosed or treated for cancer between September 2002 and June 2004 in the participating clinics were contacted.

Eligible patients were approached by a research assistant who explained the study and requested their consent to participate. Before seeing a psychologist, the consenting patients completed the HADS. At day 2 or 3 after their admission to the hospital, a structured interview was conducted by a psychologist, who was blinded to the patients' HADS scores.

The Structured Clinical Interview for DSM-IV (SCID), German Version (First *et al*, 1997; Jacobi *et al*, 2004) was conducted to act as a gold standard for identifying cases. It identifies a broad range of psychiatric disorders according to the Diagnostic Statistical Manual of Psychological Illnesses, Version 4. Beginning with screening questions, such as ‘Have you felt nervous or anxious in the last 6 months?’, the interviewer, suspecting the presence of a psychiatric disorder, continues by asking questions regarding the frequency of symptom occurrence, the extent of suffering caused by the symptoms and the general health of the patient, taking into account the possibility that the symptoms could be the results of other conditions, such as an organic illness. The study was approved by the local Ethics Committee of the University of Leipzig.

### Sample

A total of 2913 cancer patients were treated in the study clinics during the investigation period. In all, 62 (2.1%) of them could not be contacted due to early discharge or death. A total of 528 (18.1%) patients did not fulfil the inclusion criteria, resulting in 2323 remaining eligible patients. In all, 518 (38.0%) of them refused participation; 1805 (62%) patients took part in the study; and 1526 (84.5% of the study participants) were seen during the first few days following their admission to the hospital and completed the HADS questionnaire at that time. In 689 cases, a diagnostic interview was possible. Reasons for non-participation in the SCID were merely that people wanted to rest or that it was not possible to conduct the interview because of organisational problems, such as the patient being absent because of diagnostics or treatment. The resulting 689 data sets create the basis for the following analyses.

### Statistical analyses

Demographic and clinical characteristics of participating and non-participating patients were compared using *χ*^2^-tests for categorical data and the independent sample *t*-test for continuous data.

The screening ability of the HADS total and subscale scores to discriminate between cases and non-cases at a range of cutoff points was assessed using receiver operating characteristic (ROC) curves. The area under the ROC curve (AUC) indicates overall performance, with a greater AUC reflecting better performance.

The sensitivity, specificity and positive and negative predictive values (PPV and NPV) were calculated at all possible cutoff points for the HADS total and subscale scores. Sensitivity refers to the proportion of correctly identified cases and specificity to the proportion of correctly identified non-cases. Predictive value is the probability that patients identified by the HADS are cases according to the SCID; NPV refers to the proportion of true non-cases to all negative test results.

A first cutoff point (‘balanced score’) was defined so as to optimise the proportion of sensitivity and specificity scores. This proportion was calculated according to the formula (sensitivity+specificity)/2.

A second cutoff point (‘clinical score’) was set at the scores with 0.95 sensitivity, indicating a test result at which 95% of the true cases are identified by the screening instrument.

Finally, a ‘specific score’ was defined as the point at which 95% of the identified cases are true cases (=specificity 0.95 or higher).

All analyses were performed using SPSS for Windows.

## Results

### Sample characteristics

The characteristics of the study sample are displayed in Table 1. Participants who completed the SCID interview were comparable with those who did not (patients who participated in the study but were not able or willing to be interviewed) on the basis of age, tumour stage, time since diagnosis, marital status and mental well being (HADS scores). There were, however, more male SCID-participants than females (*P*<0.01).

### Psychiatric morbidity

According to the DSM-IV, 222 (32.2%) of the patients had a mental disorder. A total of 142 cancer patients (20.6%) had one, and 80 patients (11.6%) had two or more psychiatric disorders. The most frequent mental disorder was depression, with a rate of 11.6% (major depression 9.6%, dysthymia 2.0%). Of the patients studied, 6.8% had adjustment disorders, 6.4% specific phobia, 5.7% generalised anxiety disorders, 5.1% alcohol dependencies, 4.2% posttraumatic stress diseases, 3.6% (legal) drug dependencies, 1.5% (illegal) drug dependencies, 3.0% acute stress reactions, 2.9% panic disorders and 0.6% social phobia. A total of 10.4% of the patients were screened for somatisation.

In the following analyses, we deliberately did not differentiate between the different disorders. Our reasoning for this is that it is important in clinical routine to screen for every kind of mental distress and to call a psychologist to do further diagnostics and/or intervention if the screening indicates any type of disorder.

Patients with gynaecological, head and neck, urological (not prostate) and lung tumours were more affected than others, with a prevalence of 43.9, 41.3, 41.1 and 40.0%, respectively. The average HADS scores were 7.2 (s.d.: 4.2), 6.4 (s.d.: 4.5) and 13.6 (s.d.: 7.8) for the anxiety, depression and total scales, respectively.

### Optimal cutoff points

**Figure 1:**
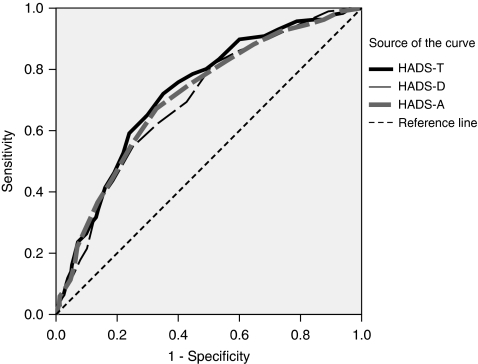
ROC curve of HADS-T, HADS-D and HADS-A. Gold standard: SCID.

The areas under the curve were 0.698 (HADS-D), 0.711 (HADS-A) and 0.726 (HADS-T) indicating a good performance of the HADS, especially the total score (see Table 2). All scales were significantly related to the gold standard criterion (all *P*-values <0.001). Figure 1 shows the ROC curves.

Different cutoff scores can be found in Table 3. The best trade between sensitivity and specificity is ⩾5 (HADS-D), ⩾7 (HADS-A) and ⩾13 (HADS-T). For clinical purposes, the following optimal thresholds were found: ⩾2 (HADS-D), ⩾3 (HADS-A) and ⩾6 (HADS-T).

## Discussion

Cancer is a threatening disease, often accompanied by difficult losses (of organ functions, social roles, physical strength, and so on), resulting in severe mental distress in a substantial number of patients (Keller *et al*, 2004; Singer *et al*, 2007). In an ideal world, every patient could see a psycho-oncologist. In reality, however, the financial resources are not there to support such an endeavour. As a result, all cancer patients should be triaged using screening instruments.

With this study, we aimed to identify optimal cutoff scores for one of the most commonly used screening tools for mental distress in oncology, the HADS.

As suggested by Morse *et al* (2005), lower thresholds should be used for cancer patients. The best trade between sensitivity and specificity for the total scale was a ⩾13 score, for example, instead of the ⩾16 score recommended by Zigmond and Snaith (1983).

What we need to think about now is what the optimal cutoff point should be for everyday clinical use. One could say that the combined level of sensitivity and specificity should be as high as possible. This would be a technical solution, potentially useful in large epidemiological studies in which it is not possible to ‘refine’ diagnostic procedures for patients with high scores, for instance, when clinical interviews are not being used. In caring for cancer patients, however, we need – in our view – other criteria. For example, it should be judged as suboptimal practice if depression in a patient who could be helped by talking with a psychotherapist or by receiving antidepressants goes undetected by the HADS and/or medical staff (depressed patients are often not identified by physicians) and remains untreated as a result. Therefore, the threshold of the HADS must be lowered. Of course, taking this approach means that specificity becomes worse, resulting in psycho-oncologists having to talk with ‘non-cases’ more often. But, is the impact of that ‘error’ comparable with the first one? We think it is not, and we would welcome a debate on this issue. We would like to argue that interaction with a patient is never a ‘waste of time’. The interaction may be brief and the patient may opt to act on an ‘offer of services’ several months or years later. One of the participants in our study wrote on the questionnaire: ‘It's good to know that I can have some support. I do not need it now but I would like to keep this option open. I might call you later.’ This is a good example for what we call prevention.

There are several limitations of our investigation that we would like to mention. First of all, it was only possible to clinically interview a portion of the patients. This was mainly due to organisational problems, but it could have resulted in a biased sample, representing only patients with few psychological problems. As, however, the prevalence rate of psychiatric disorders is comparable to similar studies (Keller *et al*, 2004), and the HADS-scores of participants and non-participants are equal, we can assume that this is not the case.

Secondly, we have to think about the timing of the administration of the HADS. In our study, the participants completed the questionnaire and the SCID soon after the admission to the hospital. It may be useful to readminister it later on. A subsample of the patients (*n*=389) in this study completed the HADS a second time before discharge from the hospital and, on an average, the mean score did indeed decrease over time (13.1 at admission, 11.1 at discharge; paired *t*-test (*t*=6.51, *P*<0.001)). However, we do not have a second SCID of all the patients and therefore cannot perform a second ROC analysis.

Nonetheless, it is more important in our view to triage the patients at the beginning of their stay in the hospital, as this is the time of the highest distress and the greatest need for support.

Another limitation is that we did not compare different screening tools, something that would have been interesting to do. Other instruments include questions regarding the need for additional information about cancer diagnosis and treatment (Herschbach *et al*, 2004; Arraras *et al*, 2007). The HADS is able to screen mental distress, but we cannot conclude from our data that it is sufficient for identifying the need for psychosocial support. This need not only depends on psychological comorbidity, but also on poor social support, and the patients' desire for such support as well (Söllner *et al*, 2001). However, knowledge and awareness of patients' level of distress is an important issue in clinical care, and the use of the HADS screening instrument can be helpful in identifying comorbid patients.

Our recommendation for clinical purposes, derived from the study results, is to use the total scale of the HADS, because it has the best AUC parameters, and it is easier to handle one scale than two. If one wants to minimise misclassifications, every patient who scores ⩾13 should be referred to a psycho-oncologist. In our dataset, 51% of all patients fulfilled that criterion. If one prefers to identify nearly all cancer patients with a mental disorder, the cutoff point should be set at ⩾6. In this study, 84% of all cancer patients had HADS scores according to that criterion. As a consequence, psycho-oncological services at many hospitals providing cancer treatment may need more financial resources than they currently have. Using a screening instrument on the other hand might, by triaging all cancer patients, help in allocating financial resources more precisely and, therefore, more adequately.

We would welcome the day when, as part of standard care, all cancer patients receive a psychosocial screening instrument, the results of which would be perused by the appropriate clinician, just as currently a standard serum test is quickly reviewed for abnormalities. In fact, this is already being done in some places and seems to work well (Sellick and Edwardson, 2007). Some clinicians might worry that patients may be bothered by there being so many questions to answer (14). We asked the participants at the end of the survey to openly express their opinion about the questionnaire. Most of the patients said they were thankful for the opportunity to answer the questions. Some patients, for example, said: ‘Thank you for this questionnaire. It helped me to think about myself and to organise my thoughts.’and ‘It was good to be able to talk about this without time pressure.’ Only two patients thought that the questionnaire was too long. Some said they would like to be examined a second time, as their cancer diagnosis was not clear yet, for example: ‘It is difficult to answer. The results of my surgery are still unclear.’ Some even articulated a wish for more in-depth conversation: ‘The results can mislead you as my feelings and my ‘life’ can not be squeezed into a matrix. I often had to add explanations as my ‘cross’ actually belonged somewhere in between the given marks.’ This last point raises again the question of how a screening instrument should best be administered and interpreted. On the basis of our clinical experiences and the responses of the participants, we think that the HADS should be handed out personally and used as a tool in the hands of experienced psychosocial clinicians.

## Figures and Tables

**Table 1:** Sample characteristics

	**N**	**%**
*Sex*		
Male	404	58.6
Female	285	41.4
		
*Age*		
<30	13	1.9
30–39	34	4.9
40–49	101	14.7
50–59	159	23.1
60–69	239	34.7
70–79	125	18.1
⩾80	18	2.6
		
*Treatment*		
Surgery	395	57.3
Chemotherapy	28	4.1
Radiotherapy	118	17.1
Radiochemotherapy	103	14.9
Diagnostics	31	4.5
Other	14	2
		
*Tumour stage (UICC)*		
0	13	1.9
I	122	17.7
II	148	21.5
III	124	18
IV	71	10.3
Not yet known	211	30.6
		
*Tumour localisation*		
Breast	76	11
Gynaecological tumours	98	14.2
Prostate	119	17.3
Other urological tumours	90	13.1
Lung	25	3.6
Colon	30	4.4
Other gastrointestinal tumours	134	19.4
Head and neck	46	6.7
Brain	35	5.1
Other	36	5.2

**Table 2:** Areas under the curve of the HADS subscales and total score

				**Asymptotic 95% confidence interval**	
	**Area**	**s.e.**	**Asymptotic significance**	**Lower bound**	**Upper bound**
HADS depression	0.698	0.023	<0.001	0.654	0.743
HADS anxiety	0.711	0.023	<0.001	0.666	0.755
HADS total score	0.726	0.022	<0.001	0.683	0.769

**Table 3:** Cutoff scores of the HADS subscales and total score

	**Score**	**Sensitivity**	**Specificity**	**(Sensitivity+specificity)/2**	**PPV**	**NPV**
*HADS Depression*						
Clinical	⩾2	0.95	0.21	0.58	0.35	0.89
Regular ZS	⩾8	0.55	0.75	0.65	0.50	0.78
Balanced	⩾5	0.82	0.49	0.65	0.42	0.85
Specific ZS	⩾11	0.30	0.88	0.59	0.53	0.73
Specific	⩾15	0.10	0.96	0.53	0.51	0.70
						
*HADS anxiety*						
Clinical	⩾3	0.95	0.18	0.56	0.35	0.88
Regular ZS	⩾8	0.67	0.67	0.66	0.48	0.82
Balanced	⩾7	0.75	0.56	0.67	0.44	0.83
Specific ZS	⩾11	0.37	0.86	0.62	0.55	0.75
Specific	⩾15	0.11	0.95	0.53	0.53	0.70
						
*HADS total score*						
Clinical	⩾6	0.96	0.21	0.59	0.36	0.91
Regular ZS	⩾16	0.59	0.76	0.67	0.53	0.80
Balanced	⩾13	0.76	0.60	0.68	0.47	0.84
Specific ZS	⩾22	0.30	0.89	0.59	0.54	0.73
Specific	⩾27	0.14	0.95	0.55	0.57	0.71

Balanced=best trade between sensitivity and specificity; Clinical=recommended score for clinical purposes, that is, sensitivity min 0.95; Regular ZS=optimal score in identifying suspicious cases according to Zigmond and Snaith; Specific=specificity min 0.95; Specific ZS=optimal score in identifying safe cases according to Zigmond and Snaith; NPV=negative predictive value; PPV=positive predictive value.
